# A National Programme for Addressing and Mitigating the Mental Health Impacts of Climate Change

**DOI:** 10.1192/j.eurpsy.2026.11105

**Published:** 2026-07-31

**Authors:** M. Gawrych

**Affiliations:** Psychology Institute, The Maria Grzegorzewska University, Warsaw, Poland

## Abstract

**Introduction:**

Terms such as climate emotions, eco-anxiety, climate grief, climate actions, heat-related mental health effects, and climate-related suicide risk frequently appear in scientific literature and health organisation guidelines.

The PubMed database contains over 1,000 articles published between 2010 and 2025 addressing the impact of climate change on mental health, including concepts such as eco-anxiety, climate grief, heat-related mental health effects, and climate-related suicide risk. A search for the phrase ‘climate change and mental health’ yields approximately 1,200 results within this period. These include systematic reviews, primary research studies, review articles, and position statements from health organisations.

**Objectives:**

The aim of this study is to identify gaps and challenges in understanding the impact of climate change on mental health, and to propose research and conceptual directions that could help to unify this scientific field.

**Methods:**

Meta-analyses and reviews from the past decade focusing on climate change and mental health were analysed. On this basis, key guidelines were developed targeting both the general population and specific groups, including climate migrants, climate activists and scientists, as well as children and adolescents

**Results:**

The results will be presented in detail during the upcoming conference.

**Conclusions:**

Developing a comprehensive strategy to prevent and mitigate the mental health impacts of climate change is a critical national priority. Adaptive and mitigative frameworks are essential for population-level prevention, addressing both direct and indirect effects through evidence-based interventions that enhance resilience and reduce vulnerability.

**Disclosure of Interest:**

None Declared

